# Multiple Zoonotic Parasites Identified in Dog Feces Collected in Ponte de Lima, Portugal — A Potential Threat to Human Health

**DOI:** 10.3390/ijerph110909050

**Published:** 2014-09-01

**Authors:** Teresa Letra Mateus, António Castro, João Niza Ribeiro, Madalena Vieira-Pinto

**Affiliations:** 1 Veterinary Medicine Department, Vasco da Gama University School, Av. José R. Sousa Fernandes, Campus Universitário—Bloco B, Lordemão, 3020-210 Coimbra, Portugal; 2 Animal and Veterinary Research Centre, University of Trás-os-Montes and Alto Douro, Quinta dos Prados, 5000-801 Vila Real, Portugal; E-Mail: mmvpinto@utad.pt; 3 Institute of Biomedical Sciences Abel Salazar, University of Porto, Rua de Jorge Viterbo Ferreira, 228, 4050-313 Porto, Portugal; E-Mail: nizaribeiro@gmail.com; 4 EpiUnit, Epidemiology Research Unit, Institute of Public Health of the University of Porto, Rua das Taipas, nº 135, 4050-600 Porto, Portugal; 5 ICETA/CECA, University of Porto, Public Health Centre Dr. Gonçalves Ferreira, National Institute of Health, Rua Alexandre Herculano, 321, 4000-055 Porto, Portugal; E-Mail: antonio.castro@insa.min-saude.pt

**Keywords:** dog, *Echinococcus*, fecal environmental contamination, parasitic zoonoses, helminths, public health

## Abstract

Dogs play many roles and their presence within people’s houses has increased. In rural settings dog faeces are not removed from the streets, representing an environmental pollution factor. Our aim was to evaluate the occurrence of environmental contamination with zoonotic intestinal parasites of three groups of dogs in Ponte de Lima, Portugal, with a particular emphasis on *Echinococcus granulosus*. We collected 592 dog faecal samples from the environment, farm and hunting dogs. Qualitative flotation coprological analysis was performed and the frequency in the positive samples ranged between 57.44% and 81.19% in different groups. We isolated up to four different parasites in one sample and detected seven intestinal parasitic species, genera or families overall. Ancylostomatidae was the most prevalent parasite, followed by *Trichuris* spp., *Toxocara* spp., *Isospora* spp., *Dipylidium caninum*, Taeniidae and *Toxascaris leonina*. Taeniidae eggs were analyzed with the PCR technique and revealed not to be from *Echinococcus*. The parasite prevalence and the diversity of zoonotic parasites found were high, which calls for a greater awareness of the problem among the population, especially hunters. Promoting research at the local level is important to plan control strategies. Health education should be developed with regard to farmers and hunters, and a closer collaboration between researchers, practitioners and public health authorities is needed.

## 1. Introduction

In human society, dogs play many roles such as pets, guarding, hunting and farming, and they are also used in therapeutic programs, life-saving actions, transport, and, last but not least, for fun and research [1]. There is evidence of the role of dogs in physical and psychological human well-being [2]. As communities become more urban, the presence of pets within houses has increased in popularity [3]. Nonetheless, dogs may represent a potential risk for human health due to the possibility of the transmission of zoonosis [4]. In urban settings, where the number of domestic animals has been increasing, dog feces represent an important pollution factor, as they are not regularly removed. Moreover, vehicular traffic, as well as the wind, can help spread viable pathogens present in dog feces, contaminating food which may later be a source of infection [5]. Parasite eggs can also be carried into human houses if adhered to shoes or animals’ paws [6]. Additionally, arthropods and other environmental factors, as the rain, may also play an important role in this context.

Current information on regional prevalence is essential to develop and modify of control measures in animal and public health [7,8]. In the Mediterranean countries, it is consensual that there is a lack of data on the prevalence of zoonotic diseases (both in animals and human), which prevents an effective epidemiological surveillance and leads to the failure in prevention. There is also an absence of intersectoral work and communication between human and animal health professionals, as well as a lack of knowledge of the human population [9]. Crucially, a relatively small number of reports have focused on the infection risk of populations living in rural or suburban settings [10], where the majority of dogs defecate in the countryside [11]. Many of these communities have large populations of free roaming domestic dogs and little access to veterinary care. These dogs have frequent contact with other animals, their feces, and a variety of refuse and foodstuffs that potentially contain zoonotic agents, which promotes infection with a variety of zoonotic agents and subsequent human exposure [12]. The aim of this study was to evaluate the occurrence of zoonotic intestinal parasites of dogs from different groups in the rural municipality of Ponte de Lima, in the northwest of Portugal, with a particular emphasis on *Echinococcus granulosus*.

## 2. Materials and Methods

### 2.1. Study Area

**Figure 1:**
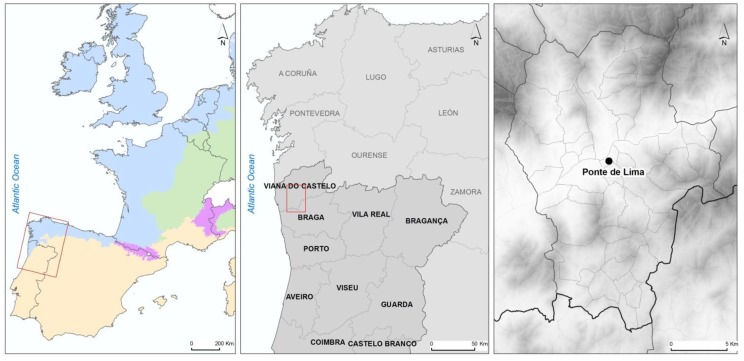
Maps of the sampling area—Ponte de Lima in the northwest of Portugal.

Most of the studies performed in Portugal concerning intestinal parasites of dogs have focused on the Centre and South of the country. The municipality of Ponte de Lima was selected for this study because it is in the North and, also, because it is a mainly rural one. The municipality belongs to the District of Viana do Castelo, and is located in the northwest of Portugal (Figure 1).

The main town in this municipality is Ponte de Lima and the region has 43,498 inhabitants dispersed over 320.26 km^2^ and 51 civil parishes [13]. The main source of income in this rural municipality is agriculture and animal breeding, on relatively small, family size farms, producing mostly corn, potatoes, wine, dairy and meat cows, sheep, goats, pigs and poultry. The strong tourism development of the region has led to an increase in the human population, especially during the summer months, and, consequently, to an increase in the urbanization of rural areas, transforming the environment and creating favorable conditions for contacts between human beings and dogs. Dog ownership is very common in Ponte de Lima. In this municipality, in 2011, there were 796 ruminant farms and between 1100 and 1500 dogs registered (official data from veterinary services). Many owned dogs have free access to the countryside. In this area, many dogs are not officially registered, so the real size of dog population is unknown.

### 2.2. Fecal Sample Collection

**Figure 2:**
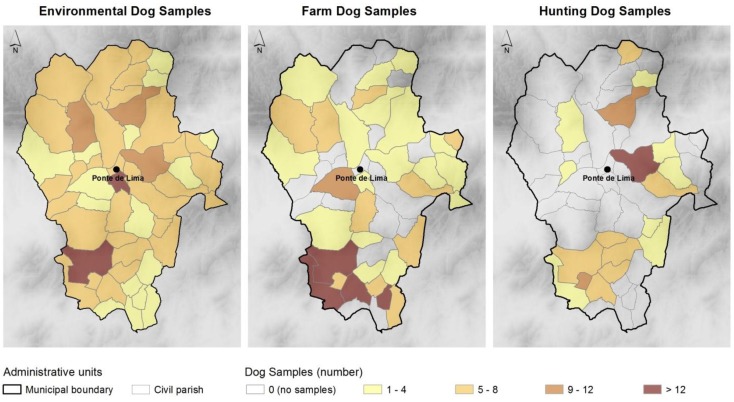
Number of samples collected per group of dogs and civil parishes.

Between December 2011 and November 2012, we collected dog feces from three different sample groups: environmental samples, farm and hunting dogs (Figure 2). Samples were randomly collected in public areas (preferably in areas attended by people, like green parks and others), ruminant farms and hunting dog packs, and are from different breeds and ages (not registered).

Fecal samples were collected fresh from the grounds in kennels, pastures or other non-specific sites. To collect environmental samples, we have walked across all the 51 civil parishes of this municipality. Three hundred ruminant farms were visited with the purpose of collecting farm dog samples, and we have collected samples in 141 of these farms. Whenever there was more than one dog in each farm, we collected more than one sample. The hunting dog samples were collected in wild boar hunting campaigns. We collected samples from 24 packs of hounds. Whenever possible, fecal samples were collected immediately after spontaneous elimination. These samples were placed in plastic containers, all individually identified, stored at 4 °C and processed within 48 h through coprological methods.

### 2.3. Coprological Methods Used

#### 2.3.1. Qualitative Flotation Coprological Analysis

Each sample was first examined macroscopically for the possible detection of proglottids. Qualitative flotation coprological analysis was performed, as described in the literature [14]. Egg identification was based on morphological characteristics (shape and structure of shell) and measurements [14,15,16]. With the exception of *Toxascaris leonina* and *Dipylidium caninum*, isolated parasites were identified at the family/genus level. A dog was classified as positive if at least one dog parasite egg, oocyst, cyst or proglottid was observed, regardless of whether or not it was zoonotic.

#### 2.3.2. Percoll Fractionation of Fecal Samples

Samples positive for Taeniidae eggs went through Percoll fractionation to render easier the molecular analysis to evaluate the presence of *Echinococcus granulosus*. Percoll (ref. 17-0891-01, GE Healthcare, Amersham Biosciences Limited, Amersham, UK) step gradients were used to perform a partial clean-up and concentration of parasite eggs. Percoll step gradients were based on the density of helminth eggs. The evaluation of Percoll fractionation was performed as described by Cardoso *et al.* [17].

#### 2.3.3. Molecular Detection of *Echinococcus granulosus*

Fecal material isolated through Percoll feces gradient was used to further evaluate the presence of *Echinococcus granulosus* in samples positive for Taeniidae. To extract the total DNA, the QIAamp ADN Mini Kit (Qiagen, GmbH, Hilden, Germany) was used according to the protocol provided by the manufacturer for the purification of DNA from tissues. The presence of *Echinococcus granulosus* DNA in fecal samples was evaluated by using PCR. For each sample we performed PCR reactions for the amplification of an *Echinococcus granulosus* repeated sequence [18]. The result was analyzed using electrophoresis. DNA samples obtained from *Echinococcus granulosus* cysts and from feces positive for *Echinococcus granulosus* were used as positive controls. These controls were carried out using PCR followed by sequencing of amplified DNA fragments.

### 2.4. Data Analysis

Results were entered into a SPSS 22.0 database. We defined prevalence as the percentage of fecal samples positive for any parasite species, and the specific prevalence as the percentage of fecal samples positive for a given parasite species. Prevalence data from all the samples were stratified into three different groups of dogs defined above: environmental samples, farm and hunting dogs. The chi-square test was used to assess the differences in proportions. A Pareto analysis was performed to describe the relative importance of each parasite species in the overall samples and after stratification. The strata were specified according to the number of parasites found simultaneously in each sample: strata 1 for the samples with one parasitic form, strata 2 for the samples with two different parasites, and so on up to strata 4 with four different forms. To assess the possible role of each sample group in the level of parasitism (independent variable), a binomial logistic regression was used to calculate the odds (OR) of having there being or not a parasitic infection in dogs in each of the three sample group, with a confidence interval (CI) of 95%. For the purposes of the binomial regression, the response variable—level of parasitism—was categorized into two categories: no parasites in feces and parasite in feces. To go further and assess the influence of each of the three sample groups of the level of parasitism in dog feces (independent variable), a nominal logistic regression analysis was used to calculate the OR. The response variable was split into three levels: level zero with no parasitic forms, level one for samples showing one parasitic species and level two for fecal samples showing two of more parasitic species. A CI of 95% was used.

## 3. Results

### 3.1. Risk of Infection by Strata

A total of 592 samples were collected from three different groups of fecal samples. Out of these dog fecal samples, 374 were positive for the presence of parasitic forms. The prevalence of parasites found in the three groups of dog fecal samples is presented in Table 1.

**Table 1:** Prevalence of parasites found in dog fecal samples collected in Ponte de Lima, Portugal.

Presence of parasite	Environmental Dog Samples (*n* = 296)		Farm Dog Samples (*n* = 195)		Hunting Dog Samples (*n* = 101)		Total (*n* = 592)	
	*n*	%	*n*	%	*n*	%	*n*	%
Negative	119	40.20	83	42.56	19	18.81	218	36.82
Positive	177	59.80	112	57.44	82	81.19	374	63.17

### 3.2. Diversity of Parasites Found and Individual Prevalence

In the 592 samples, seven species/genera/families of intestinal parasites were detected from Classes Nematoda, Cestoda (although proglottides were not identified during these procedures) and Coccidia (Table 2).

**Table 2:** Prevalence of parasitic species found in 592 dog fecal samples from three different groups.

Parasite	Environmental Dog Samples (*n* = 296) (%)	Farm Dog Samples (*n* = 195) (%)	Hunting Dog Samples (*n* = 101) (%)
Ancylostomatidae	44.59	31.28	70.30
*Trichuris* spp.	34.46	32.82	49.50
*Toxocara* spp.	7.43	11.28	10.89
*Toxascaris* *leonina*	0.68	0	0
*Dipylidium caninum*	0.68	1.02	0.99
Taeniidae	0.34	0.51	1.98
*Isospora* spp.	3.04	1.54	4.95

**Figure 3:**
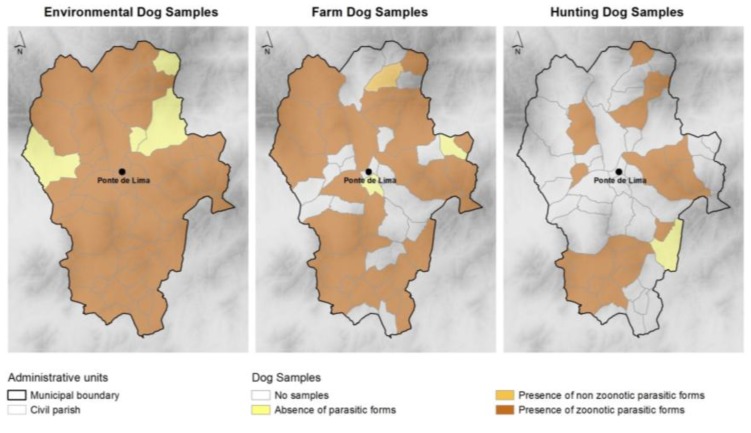
Location of samples without and with (zoonotic and non-zoonotic) parasitic forms per dog group and civil parishes.

Taeniidae eggs were present in four samples. These samples were analyzed with the polymerase chain reaction technique, which revealed the eggs to be from *Taenia* spp. and not *Echinococcus granulosus*. These eggs were more frequent in hunting dog samples. The distribution of zoonotic (Ancylostomatidae, *Trichuris* spp., *Toxocara* spp., *Dipylidium caninum* and Taeniidae) and non-zoonotic parasites throughout the municipality is shown in Figure 3.

### 3.3. Parasite Associations and Infections

The presence of zero, one, two or more parasitic species in the fecal samples examined is summarized in Table 3.

**Table 3:** Presence of zero, one and two or more parasitic species in 592 dog fecal samples examined.

Number of Different Parasites	Environmental Dog Samples (*n* = 296) (%)	Farm Dog Samples (*n* = 195) (%)	Hunting Dog Samples (*n* = 101) (%)
0	40.2 ^a^	42.6 ^a^	18.8 ^a^
1	33.4 ^a^	39.0 ^a^	30.7 ^b^
>1	26.4 ^a^	18.5 ^b^	50.5 ^c^

^a,b,c^ Each subscript letter indicates a subset of categories whose proportions on columns; did not differ significantly from each other at the, 0.05 level.

The parasites and association of parasites found in three different groups of dog fecal samples is presented in Table 4.

**Table 4:** Parasites and association of parasites found in three different groups of dog fecal samples.

Parasite	Environmental Dog Samples (*n* = 296) (%)	Farm Dog Samples (*n* = 195) (%)	Hunting Dog Samples (*n* = 101) (%)
Ancylostomatidae	19.59	14.87	20.79
*Trichuris* spp.	11.49	16.41	5.94
*Toxocara* spp.	1.35	5.64	2.97
*Dipylidium* *caninum*	0.38	0	0.99
*Toxascaris leonina*	0.38	0	0
*Isospora* spp.	0.38	0	0
Taeniidae	0	0.51	0
Ancylostomatidae + *Trichuris* spp.	17.91	10.77	34.65
Ancylostomatidae + *Toxascaris leonina*	0	0	0.99
Ancylostomatidae + *Toxocara* spp.	2.03	2.56	2.97
*Trichuris* spp.* + Toxocara* spp.	1.01	2.56	0.99
Ancylostomatidae* + Dipylidium* *caninum*	0.38	0.51	0.99
Ancylostomatidae + *Isospora* spp.	0.38	0	0
Ancylostomatidae + *Trichuris* spp. + *Toxocara* spp.	1.69	0.51	1.98
Ancylostomatidae + *Trichuris* spp. + Taeniidae	0	0	0.99
Ancylostomatidae + *Toxocara* spp. + Taeniidae	0	0	0.99
Ancylostomatidae + *Trichuris* spp. + *Toxascaris leonina*	0	0	1.98
Ancylostomatidae + *Trichuris* spp. + *Isospora* spp.	1.35	1.54	1.98
Ancylostomatidae + *Isospora* spp. + *Toxocara* spp.	0.38	0	0
*Trichuris* spp. + *Toxocara* spp. + Taeniidae	0.38	0	0
*Dipylidiumcaninum* + *Toxocara* spp. + *Trichuris* spp.	0	0.51	0
Ancylostomatidae + *Isospora* spp. + *Toxocara* spp. + *Trichuris* spp.	0.38	0	0.99
Ancylostomatidae + *Isospora* spp. + *Toxocara* spp. + *Toxascaris leonina*	0.38	0	0.99

**Figure 4:**
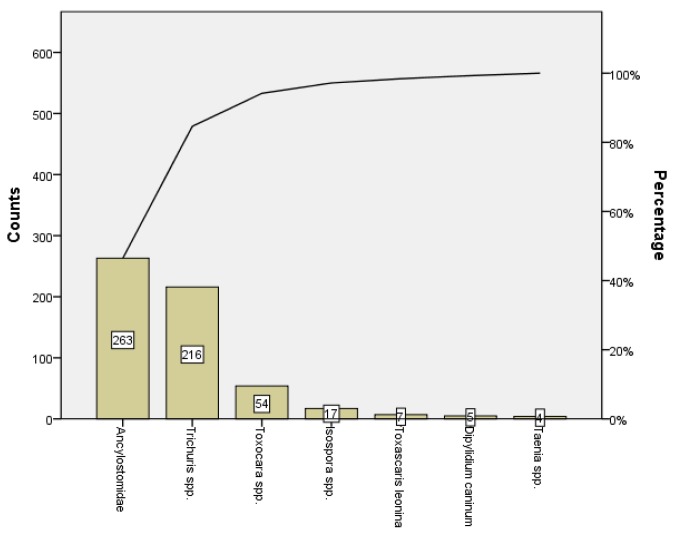
Pareto analysis of the occurrence of parasitic forms.

A Pareto analysis of the occurrence in samples of parasitic forms was performed and showed that 84.6% (479/566) of the parasitic forms were Ancylostomatidae and *Trichuris* spp (Figure 4). 

Logistic regression analyses were performed. The binomial logistic regression showed that environmental and farm dog samples are not different with regard to risk factors for parasitism (*p* = 0.603). Nonetheless, the risk of parasitism occurrence is 2.9 times higher in hunting than in environmental dog fecal samples: OR 2.9 (1.7–5.0); *p* < 0.000. As for the nominal logistic regression analysis, hunting dogs was the reference category and two different levels of parasitism were assessed against the no parasite category. The risk of being infected with a single infection, does not differ between the three groups. Regarding the existence of multiple infections (two or more different parasites in the same sample), the risk is significantly higher (OR: 4.1) for hunting dogs (*p* < 0.000) than for environmental dog samples and also for hunting dogs (OR: 6.2) than for farm dogs (*p* < 0.000).

## 4. Discussion

### 4.1. Risk of Infection by Group

The prevalence of parasites found in dog fecal samples in Portugal and in other countries are presented in Table 5. 

**Table 5:** Prevalence of parasites found in dog fecal samples in Portugal and in other countries.

Authors	Country	*N*	Dog Sample Origin	Overall Prevalence
Tarsitano* et al.* [5]	Italy	152	Environmental	8.5%
Rinaldi* et al.* [19]	Italy	415	Environmental	16.9%
Dubná* et al.* [20]	Czech Republic	3780	Environmental + Shelter Dogs	17.6%
Martínez-Carrasco* et al.* [21]	Spain	275	Dogs presented to veterinary clinics + Shelter Dogs + Stray Dogs	25.0%
Papazahariadou* et al.* [22]	Greece	281	Farm Dogs + Hunting Dogs	26.0%
Gracenea* et al.* [23]	Spain	505	Shelter Dogs	26.9%
Soriano* et al.* [24]	Argentina	1944	Environmental	37.9%
Szabová* et al.* [1]	Slovak Republic	752	Environmental + Owned Dogs + Shelter Dogs	45.7%
Balassiano* et al.* [25]	Brazil	500	Dogs presented to veterinary clinics	46.4%
Fontanarrosa* et al.* [7]	Argentina	2193	Owned Dogs	52.4%
Okoye* et al.* [26]	Nigeria	413	Stray Dogs	52.6%
Benito* et al.* [27]	Spain	1040	Shelter Dogs	53.6%
Katagiri and Oliveira-Sequeira [28]	Brazil	254	Owned Dogs	54.3%
Beiromvand* et al.* [29]	Iran	77	Owned Dogs + Stray Dogs	66.0%
Bajer* et al.* [30]	Poland	108	Sled Dogs	68.0%
Ugbomoiko* et al.* [31]	Nigeria	396	Owned Dogs	68.4%
Martínez-Moreno* et al.* [32]	Spain	1800	Shelter Dogs	71.3%
Gingrich* et al.* [33]	Galapagos Island	97	Owned Dogs	71.4%
Eguía-Aguilar* et al.* [34]	México	120	Stray Dogs	85.0%
Mandarino-Pereira* et al.* [35]	Brazil	81	Environmental	92.6%
Crespo and Jorge [36]	Portugal	576	Environmental	17.9%
Cruz* et al.* [37]	Portugal	49	Environmental	18.4%
Neves* et al.* [38]	Portugal	368	Dogs presented to veterinary clinics	20.6%
Mateus* et al.* [39]	Portugal	100	Shelter Dogs	41.0%
Crespo* et al.* [40]	Portugal	548	Environmental	50.0%
Cardoso* et al.* [17]	Portugal	301	Farm Dogs	58.8%

The diversity of results obtained from different studies highlights the importance of promoting research at the local level to plan control strategies [7]. The differences reflected in Table 5 may be partially explained by the origin of the dog samples. Animals placed in shelters are dewormed by veterinarians [41], but the high densities observed frequently in shelters may contribute to the propagation of these parasites [42]. There is also a wide disparity with regard to the sampling collection methods (many of the studies included in Table 5 involved collection of samples *per rectum* or in necropsy), the number of samples collected and the coprological method used, making it difficult to compare results. There is no unique technique capable to do the parasitological diagnosis of all kinds of parasitic species that may be present in feces [43]. Although compared to *post-mortem* fecal examination fecal flotation is less sensitive in the detection of parasites [21,44], faecal flotation is considered a very valuable method for the assessment of the majority of dog parasites [45]. The frequency found in the positive samples is high, and the risk of parasitism and occurrence of multiple infections is higher in hunting dogs. This highlights the need of health education in a specific target group: hunters and their families.

A much lower parasite prevalence in hunting dog samples than in the present study has been previously reported [22]. Hunting dogs are at the highest risk of worm infections and are therefore responsible for most environmental contamination and human disease [46]. Helminth zoonoses transmitted among dogs, wildlife, and people have been discussed by Jenkins* et al.* [47]. In Portugal the impact of wildlife on public health is unknown [17], however, zoonotic helminths in wild carnivores fecal samples have been found in footpaths of a protected area in Ponte de Lima, where people, domestic and wild animals coexist [48].

Access to soil, the hygiene of the environment, illness, the owners’ level of education, and veterinary care, are all associated with intestinal parasite infections in dogs [25]. Concerning the farm and hunting dogs that were sampled, no clinical signs were observed, this is in agreement with Neves* et al.* [38]. In a study about dog owners’ awareness conducted in this municipality it was concluded that the practices used to deworm dogs, if any, were not correct, and few owners referred fecal elimination after deworming [49], which is in agreement with other surveys [6,22]. Szabová* et al.* [1] found that the environmental samples had the lowest prevalence recorded. In our study, some of the environmental samples were not as fresh as those from the farm or hunting dogs, so it was somehow expected to find lower parasite prevalence in these samples. On the other hand, supposing that environmental samples correspond to stray dogs, these usually do not undergo deworming, and therefore they are possible carriers of many parasites [32,35]. Our results could be explained by our environmental samples in fact not being from homeless dogs, but from owned dogs that have free outside access, as Ponte de Lima is mainly a rural municipality.

### 4.2. Diversity of Parasites Found and Individual Prevalence

In the examined samples, seven parasitic species were isolated. A similar number has been recorded in other studies [17,21,41], although several refer a higher [1,7,20,22,23,24,25,28,29,34,43,50,51] or lower [26,30,31,33,35,36,52] number of parasitic species. In addition to the number of parasitic species, there is a great variability in isolated species and specific prevalence which indicates the necessity of being cautious when extrapolating conclusions from data from one location to another.

There is also variety concerning the most prevalent intestinal parasite found in different studies: Ancylostomatidae or *Ancylostoma caninum* [7,17,26,28,30,32,33,34,35,36,43,50,52], *Toxocara* spp. or *Toxocara canis* [1,5,22,24,51,53], *Isospora canis* [38], *Giardia* spp. [41,54] and *Cryptosporidium* spp. [25]. Once again this discrepancy may be due to most of the variables previously mentioned, namely the different methodologies used.

A Pareto analysis showed that most of the parasitic forms were Ancylostomatidae and *Trichuris* spp. Interestingly Rubel* et al.* [55] reported that the highest prevalence of eggs of both these parasite was detected in areas with lower socio-economic level, and Szabová* et al.* [1] pointed to the contamination of the environment in which animals move.

For Papazahariadou* et al.* [22], Ancylostomatidae were more frequent in farm dogs rather than hunting dogs, unlike in our study.This may be associated, in our study, with the local habit of keeping hunting dogs in kennels where they defecate, maintaining a highly infective habitat [11]. Dogs are hosts to hookworms that may cause zoonotic diseases, most notably *cutaneous larva migrans* [56].

*Trichuris* spp. were the most frequent intestinal parasites found in hunting dogs. Most of the other studies have reported a much lower prevalence [1,17,20,24,26,38,53,54]. The eggs may remain viable and infective in the environment for years, leading to high infection rates in dogs [57]. Humans can be infected by *Trichuris vulpis* some cases have already been reported [58]. *Toxocara* eggs were more commonly found in farm dogs. According to Cardoso* et al.* [17], the domestic slaughtering of pigs and small ruminants showed a statistical association with *Toxocara* infections, and this practice is usual in farm dog owners in this municipality. Human infection is caused by direct contact with contaminated soil or dog hair [59]. *Visceral larva migrans*, *ocular larva migrans* [60], and severe diseases affecting the central nervous system and/or the eye can occur [61]. We found infective *Toxocara* spp. eggs in a few environmental samples. In most rural and urban resource-limited communities, children are considered the highest risk group [12], however in farms, adults and children are equally susceptible to soil-transmitted infections [62]. Very high *Toxocara canis* seroprevalence has been found in farmers, veterinarians, slaughterhouse staff and hunters [63]. Health education to raise public awareness is therefore strongly encouraged [26].

*Toxascaris leonina* is an ascarid less frequent than others, as our and other studies confirm [20,24,38,41,51,53]. In contrast, Beiromvand* et al.* [29] found it to be the most frequent. *Dipylidium caninum* has been usually considered the most frequent Cestoda in dogs [27,32,50], as reported in our study. Humans can become infected and very young children are the ones most often affected [64]. Symptoms are usually absent, although abdominal discomfort, diarrhea and pruritus may be present [65].

The detection of Taeniidae eggs in fecal samples by routine microscopy suffers from low sensitivity [6]. A cross-sectional survey in Germany and other European countries have detected these eggs only in 0.25% of the samples [66]. In Portugal, owning cattle was found to be a significant risk factor for *Taenia* spp. presence in dogs [17] however, in our study, the higher prevalence of positive samples was not in farm dogs, but in hunting dogs. The coat of the foxes can be contaminated with Taeniidae eggs [67], so hunters—human and dog—can be directly exposed to these immediately infective eggs [6]. *Echinococcus* spp. eggs are morphologically indistinguishable from *Taenia* spp. Echinococcosis is one of the five most important zoonoses in the Mediterranean region [68], nonetheless, it remains a neglected zoonosis [69]. Despite the low sensitivity of fecal based methods to *Echinococcus granulosus* egg detection, it is revelant for public health to remark the absence of these parasites in our study.

Unlike in some studies [25,38,41,54], helminth eggs were more commonly identified than protozoan. The trend of reducing helminthic and increasing protozoan infection has been attributed to the knowledge of dog owners about potential zoonotic transmission of these agents and how to control them [70]. These facts stand in contrast with the results of our study, in this may be explained by limited awareness in Ponte de Lima. Although the methods used are not appropriate for protozoan diagnosis we did find *Isospora* spp. and these parasites were more prevalent in hunting dog samples.

Undoubtedly, the present study revealed poor hygiene conditions and animal housing, similarly to Cardoso* et al.* [17]. Furthermore, a wide diversity of zoonotic parasites has been detected, and well distributed throughout the municipality.

### 4.3. Parasite Associations and Infections

The majority of the dogs were infected by only one species of parasite. Similar results have been reached by other studies, but with a distinct number of different parasites found: up to two [38], up to three [21,26,30,32,35,36,41], up to six [51] and up to seven [1]. Hunting dogs had a significantly larger risk of being infected with multiple parasitic species.The most frequent association observed between parasites was Ancylostomatidae and *Trichuris* spp. A subsequent Pareto analysis, carried out by strata confirmed the predominance of these two parasites. Interestingly, the same association has also been found between human whipworms and hookworms [71]. It is still not clear what drives this affinity between parasites. Answers to this question are essential to adequately understand the epidemiology and control of these diseases. These findings warrant further studies to understand whether there are biological factors influencing such co-existence [57].

### 4.4. Limitations of the Study

Our study design had some necessary limitations: the prevalence of infection in dogs could not be determined in environmental samples. Consequently although we knew the background of each individual dog from the other groups, we did not plan to trace back the samples to the animal of origin, as our aim was not to do that. We did not record breeds or ages, and with regard to environmental samples, multiple samples may have originated from a single dog, although we tried to avoid this from taking place. In our survey, the size of the dog population is expected to be large enough to draw reliable conclusions.

### 4.5. “One Health” Approach Is Required

A close collaboration between veterinary and public health professionals in a “One Health” approach is required [5,6,11,72]. In Portugal, veterinary practitioners acting as information sources about zoonoses transmitted by canids are needed, but there are basic priorities that should be considered first. So far, data is limited to only a few urban areas and no information is available for large territories of the country with more suitable socioeconomic and environmental conditions for parasitic transmission. Multidisciplinary approaches will lead to a more complete understanding of the actual epidemiological situation in the country. Few studies have been undertaken to determine the prevalence of these organisms and/or their associated diseases in people from the same community. Many physicians are not knowledgeable about these infections [73], and they feel that a collaborative relationship with a veterinarian who possesses specialty training in zoonoses would be valuable to their practice [74]. Several studies have shown that physicians delegate to veterinarians the responsibility to do health education of communities concerning zoonoses [46,74,75], and suggest that veterinarians should be involved not only in controlling zoonotic disease in animals, but also in providing information for patients and physicians [75]. Nonetheless, communication among veterinarians, physicians, and dogs owners or human patients seems to be insufficient [76]. Although changing in human behavior is an extremely difficult challenge [46], this is essential for the success of the control and prevention of these diseases. Effective campaigns and education programs could be instituted to prevent of zoonotic infections associated with household pets and address issues surrounding poorly prepared or cooked food [76].

## 5. Conclusions

Zoonoses involving dog parasites are both common and important, with some causing serious diseases. Understanding the epidemiology of zoonotic parasitic infections is important to minimize of the risk to humans. The epidemiological research conducted in Ponte de Lima revealed a considerable environmental contamination with zoonotic parasites. The two most prevalent parasites are Ancylostomatidae and *Trichuris* spp. The high level of environmental zoonotic contamination found calls for raising the awareness of the problem among the population. Hunting dogs were at higher risk of harboring zoonotic parasites showing higher prevalence and a higher number of multiple infections. Health education and risk communication should therefore be developed to and target children and families, farmers, and, especially, hunters. Also, closer collaboration is needed between researchers and practitioners (human and veterinary medical professionals), as well as with public health authorities. The prevention of zoonoses requires a global commitment, however, the main task still regards local populations and requires changes to human behavior. Population awareness is a pressing need.
